# Synergistic induction of the clock protein PERIOD by insulin-like peptide and prothoracicotropic hormone in *Rhodnius prolixus* (Hemiptera): implications for convergence of hormone signaling pathways

**DOI:** 10.3389/fphys.2014.00041

**Published:** 2014-02-19

**Authors:** Xanthe Vafopoulou, Colin G. H. Steel

**Affiliations:** Biology Department, York UniversityToronto, ON, Canada

**Keywords:** insulin signaling, PTTH signaling, daily cycling, rhythm, pigment dispersing factor, PDF, lateral neurons

## Abstract

We showed previously that release of the cerebral neurohormones, bombyxin (an insulin-like peptide, ILP) and prothoracicotropic hormone (PTTH) from the brain have strong circadian rhythms, driven by master clock cells in the brain. These neurohormone rhythms synchronize the photosensitive brain clock with the photosensitive peripheral clock in the cells of the prothoracic glands (PGs), in which both regulate steroidogenesis. Here, using immunohistochemistry and confocal laser scanning microscopy, we show these neurohormones likely act on clock cells in the brain and PGs by regulating expression of PERIOD (PER) protein. PER is severely reduced in the nuclei of all clock cells in continuous light, but on transfer of tissues to darkness *in vitro*, it is rapidly induced. A 4h pulse of either PTTH or ILPs to brain and PGs *in vitro* both rapidly and highly significantly induce PER in the nuclei of clock cells. Administration of both neurohormones together induces more PER than does either alone and even more than does transfer to darkness, at least in PG cells. These are clearly non-steroidogenic actions of these peptides. In the peripheral oscillators salivary gland (SG) and fat body cells, neither bombyxin nor PTTH nor darkness induced PER, but a combination of both bombyxin and PTTH induced PER. Thus, PTTH and ILPs exert synergistic actions on induction of PER in both clock cells and peripheral oscillators, implying their signaling pathways converge, but in different ways in different cell types. We infer clock cells are able to integrate light cycle information with internal signals from hormones.

## Introduction

In the blood sucking bug *Rhodnius prolixus*, insulin-like peptides (ILPs) are synthesized in identified neuroendocrine cells in the brain and are released into the haemolymph with a circadian rhythm in both the larval and adult stages (Vafopoulou and Steel, 2002, 2012b). A blood meal elicits rapid and dramatic release of ILPs indicating a role of ILPs in nutrient sensing (Vafopoulou and Steel, 2012b). In *Rhodnius*, bombyxin, the first ILP isolated from insects (Nagasawa et al., 1988), possesses mild ecdysteroidogenic activity on prothoracic glands (PGs) (Vafopoulou and Steel, 1997). The daily rhythm of synthesis and release of ILPs is tightly coupled to the daily rhythm of production and release of another cerebral neurohormone, the prothoracicotropic hormone, PTTH (Vafopoulou and Steel, 1996a,b). The only known function of PTTH is regulation of the synthesis of ecdysteroids by the paired PGs. PGs are photosensitive circadian clocks (Vafopoulou and Steel, 1992, 1998) which generate the circadian rhythm of ecdysteroid synthesis and release (Vafopoulou and Steel, 1991). Thus, all three hormones ILPs, PTTH and ecdysteroids are released with a synchronous daily rhythm. The circadian timing system in the brain has been described in detail (Vafopoulou et al., 2010; Vafopoulou and Steel, 2012a) and is the primary regulator of all three rhythms. Within the brain, axonal projections from the clock cells make intimate associations with both the ILP neurons (Vafopoulou and Steel, 2012b) and the PTTH neurons (Vafopoulou et al., 2007), revealing nervous pathways by which the brain timing system can drive rhythmic release of these neuropeptides. The PTTH rhythm acts on the PGs, in which it entrains the rhythm of steroidogenesis generated by the PG clock (Pelc and Steel, 1997; Vafopoulou and Steel, 1999), suggesting it may act on the clock in the PG cells. Since ILPs also possess steroidogenic activity on PGs, these too may influence the PG clock. Thus, existing evidence suggests that brain neuropeptides act as “internal messengers of time” (internal Zeitgebers). The present work provides direct evidence that both bombyxin and PTTH do indeed act on the canonical clock protein PERIOD (PER) in the molecular clock in the PGs and also in the clock cells in the brain. Their actions on PER induction are shown to be synergistic, which indicates convergence in the signaling pathways for the two peptides at some point(s).

The broader significance of clock control of rhythmicity in these hormones resides in the functional importance of the resulting circadian rhythm in circulating ecdysteroids. Ecdysteroids are of central importance in the coordination both of development (in the larva) and of reproduction (in the adult) [e.g., reviews by Marchal et al. (2010), Schwedes and Carney (2012)]. Almost all insect cells possess the ecdysteroid receptor (EcR), and we have shown that circadian cycling of EcR occurs in numerous cell types (Vafopoulou and Steel, 2006). This finding shows that many cell types respond to the rhythm of circulating ecdysteroids. We have suggested (Steel and Vafopoulou, 2006) that rhythmic ecdysteroid responses serve first, to orchestrate cellular events in target cells around a circadian cycle and second, to synchronize rhythmicities in distant target cells with each other, thereby creating internal order throughout the animal.

The present work also shows that this temporal order is not generated exclusively by ecdysteroids. We show that certain tissues possess peripheral oscillators (these do not free run in aperiodic conditions and so are driven oscillators, not clocks), specifically salivary glands (SGs) and fat body (FB), which are driven directly by ILPs and PTTH from the brain. The response to the neuropeptides also involves their action on PER in these cell types, but in a different manner from their action on the PG and brain clock cells, that is also indicative of convergence in the signaling pathways for the neurohormones.

## Materials and methods

### Animals, tissues and *in vitro* incubations

*Rhodnius* larvae were raised at 28 ± 0.5°C in 12 h light: 12 h dark (12L:12D). Only male 5th (last) instar larvae were used in experiments. Unfed larvae exist in a state of developmental arrest and larval-adult development is initiated by a large blood meal. Animals raised in 12L:12D were used as controls to determine daily cycling of PER in various cell types (see below).

Experimental animals were transferred to continuous light (LL) 3 weeks prior to feeding. This long exposure to LL abolishes all known circadian rhythms of synthesis and release of several cerebral neuropeptides and synthesis and haemolymph titer of ecdysteroids (Vafopoulou and Steel, 1992, 2001). These larvae are called LL larvae. At day 12 after feeding of LL larvae, tissues were dissected and subjected to various treatments *in vitro*. Eight animals were sacrificed per experiment. For experiments with brains, the number of brains used is stated in Results. Tissues included the brain, PGs, SGs and pieces of abdominal FB. For *in vitro* incubations of LL tissues, all tissues with a certain cell type were pooled and each tissue type was incubated individually in incubation chambers for 4 h *in vitro* in 100 μl *Rhodnius* saline (Lane et al., 1975), containing glucose and antibiotics under constant gentle agitation. Incubations were: (A) Incubation in saline in light (controls). (B) Incubation in saline in dark. (C) Incubation in the presence of crude protein extract from brains of unfed fifth instar larvae (one brain equivalent per incubation chamber); these extracts were shown previously to contain biologically active PTTH and ILPs (see Vafopoulou and Steel, 1996a, 2002, 2012a). Extraction of proteins from brains has been described before (Vafopoulou and Steel, 1996a). (D) Incubation in the presence of bombyxin. (E) Incubation in the presence of PTTH. (F) Incubation in the presence of both bombyxin and PTTH. (G) Synthetic Pigment Dispersing Factor of *Uca pugilator* (PDF) (see below) was employed as control peptide in the above experiments. All tissues were subjected simultaneously to different treatments. After termination of incubation, all tissues were processed simultaneously for immunohistochemistry.

### Antibodies, peptides, and chemicals

A rabbit polyclonal antibody to PER was used which was prepared against a 14-amino acid sequence corresponding to the protein-protein dimerization motif of the PAS region of PER (residues 605–618; KSSTETPPSYNQLN; known as peptide PER-S, bleed S80-3) of *Drosophila* and was a generous gift of Dr. Kathleen Siwicki (Swarthmore College, Swarthmore, PA). The specificity of binding of this antibody to *Drosophila* PER was demonstrated by Siwicki et al. (1988). The 14-amino acid sequence of PER-S is highly conserved among PER proteins. This antibody recognizes the native PER of *Rhodnius* (Vafopoulou et al., 2010) and was used at a dilution of 1:1000.

A guinea pig polyclonal antibody was prepared against a custom-made, synthetic peptide for the complete amino acid sequence of *Uca pugilator* pigment dispersing hormone (PDH) (NSELINSILGLPKVMDA) (GenScript, Piscataway, NJ). The insect homologs of PDH are known as pigment dispersing factors (PDFs). PDF antibodies have been used extensively to trace axonal projections of clock cells in the brains of various insects including *Rhodnius* (Vafopoulou et al., 2010; Vafopoulou and Steel, 2012a). The specificity of this antibody to recognize *Rhodnius* PDF was determined by double immunochemistry with an anti-PDF used extensively before by us (Vafopoulou et al., 2007, 2010; Vafopoulou and Steel, 2012a,b) as well by many other laboratories. This antibody was used to trace the axonal projections of the *Rhodnius* brain clock cells and produced a staining pattern that completely co-localized with the staining pattern produced by the previously used anti-PDF. This antibody was used at a dilution of 1:500.

Recombinant *Bombyx* PTTH (PTTH) (expressed in *E. coli*) (Ishibashi et al., 1994) and synthetic bombyxin-II (Nagasawa et al., 1988) were generous gifts from A. Mizoguchi (Nagoya University, Nagoya, Japan). These peptides exhibit steroidogenic activity in the PGs of *Rhodnius* (Vafopoulou and Steel, 1997) and antibodies against these peptides were used to immunologically identify the *Rhodnius* PTTH and ILPs (Vafopoulou and Steel, 2002) and to localize the neurons producing them in the brain (Vafopoulou et al., 2007; Vafopoulou and Steel, 2012b). PTTH was used at 8 ng/ml dilution and bombyxin was used at 300 ng/ml dilution in *Rhodnius* saline. These concentrations are the lowest that induce maximal stimulation of ecdysteroid synthesis by PG cells *in vitro* in published dose-response curves (Vafopoulou and Steel, 1997). For combination treatments with both bombyxin and PTTH, the peptides were used at the above concentrations. As control, synthetic PDF was used at its haemolymph concentration of 1 nM (Persson et al., 2001) and at 10 nM concentrations. No effect of PDF on PER was seen in any experiment, hence these data are not included in Results.

Goat anti-rabbit and goat anti-guinea pig IgGs conjugated to the green fluorophore fluorescein isothiocyanate (FITC) used in immunohistochemistry were purchased from Sigma–Aldrich (St. Louis, MO). Vectashield mounting medium was purchased from Vector Laboratories, Burlington ON, Canada.

### Immunohistochemistry, image collection, and statistical analyses

All tissues were prepared according to an established protocol (e.g., Vafopoulou and Steel, 2012a). Briefly, issues were fixed in freshly prepared 4% paraformaldehyde in phosphate-buffered saline (PBS, pH 7.2) for 2 h at room temperature, washed thoroughly in PBS (pH 7.2) and preincubated in 5% control serum containing 1% Triton X-100 as a permeabilizing agent. Tissues were then incubated for 24 h in solutions containing the primary antibodies at 4°C. Tissues were thoroughly washed in PBS and incubated overnight in the secondary antibody generated in goats. All secondary antibodies were used at 1:200 dilution. After thorough washing in PBS, tissues were mounted in Vectashield. In technical controls, the primary antibody was replaced with non-immune serum or the secondary antibody was replaced with PBS. Fluorescence levels in these controls were indistinguishable from the background and no autofluorescence was detected. Digital optical sections at 1 μm distances were viewed with an Olympus FV300 confocal laser scanning microscope. Microscope parameters were kept constant. Images were processed using ImageJ (1.47 q) (NIH, Bethesda, MD) and Adobe Photoshop CS5 (San Jose, CA). The digitized images were modified only to adjust contrast and merge files.

Pixel intensity of fluorescence in nuclei and cytoplasm of randomly selected incubated cells was quantified using the line tool in Image J on original images from the microscope. The length of the line was kept constant in all measurements. Mean pixel intensity was calculated for all cell types and all different treatments as follows: four cells were selected randomly from a particular tissue from a single animal and pixel intensities were measured separately for nuclei and cytoplasm. Cells were chosen using randomization procedures that vary somewhat between tissue types because structure of the tissues do not permit the same procedure for all cell types. For example, PG cells form a line along the edges of the prothoracic lobe; we picked every 4th cell in a row. Fat body cells are spread on a one-cell thick sheet; we used a grid with designated spots in which the cells were counted in each preparation. The same grid was used in all experiments. SG cells are spread as single cell sheets over the outer surface of the SG. We picked every 4th cell on a straight line longitudinally. Then, the average values from these four measurements of fluorescence intensities were separately calculated for nuclei and cytoplasm. These averages represented values corresponding to a single animal. Since eight animals were used per treatment, the mean pixel intensity (±s.e.m.) of nucleus and cytoplasm was calculated from eight averages corresponding to eight animals. This was done for each cell type and for each treatment. For brains, because 12 or 15 animals were used as brain donors, 12 or 15 such averages (see Results) were used to calculate mean pixel intensities of cytoplasmic fluorescence in LNs per treatment. Only LNs in the right optic lobe were used for measurements. Comparison between treatments of a particular cell type were made using two non-parametric tests, the Mann–Whitney *U*-test (Mann and Whitney, 1947) and the Kruskal-Wallis 3- or 4-point test (Kruskal and Wallis, 1952). Background fluorescence levels were minimal and were obtained from adjacent regions in the same preparations that lacked evident fluorescence. These values were subtracted from all measurements before statistical analysis.

## Results

### Cycling of PER in cells of entrained animals

**Figure 1:**
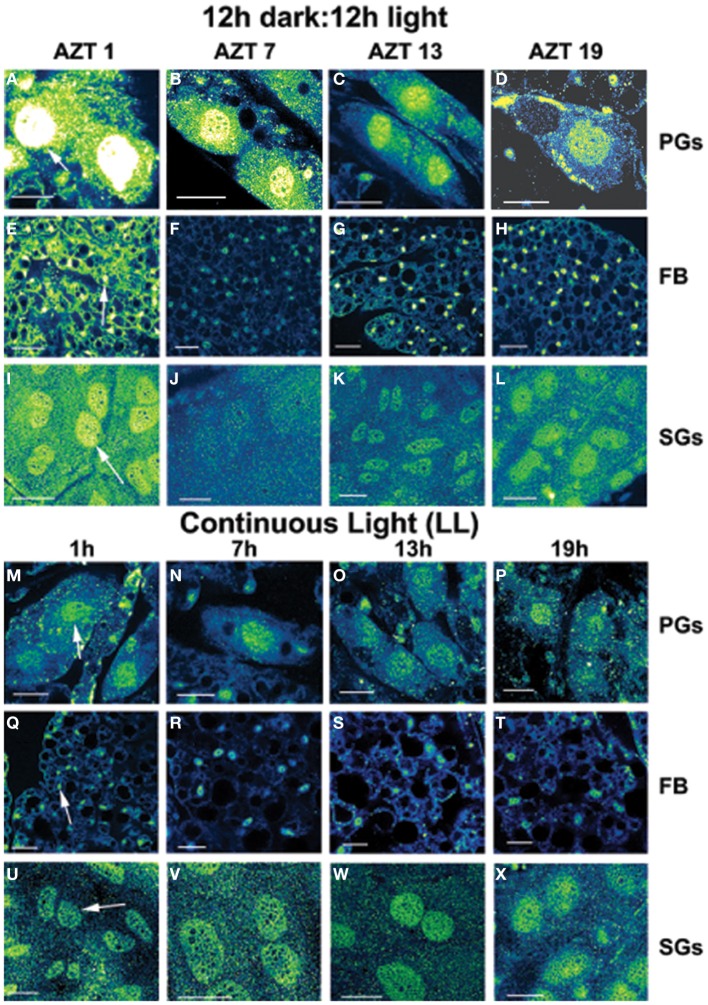
**Daily cycling *in vivo* in abundance and nuclear localization of PER immunofluorescence (A–L).** Green/yellow/white shows fluorescence. Tissue samples were dissected at four times during day 12 after a blood meal: 1 h after lights-off (AZT 1), 7 h after lights-off (AZT 7), 1 h after lights-on (AZT 13) and 7 h after lights-on (AZT 19). **(A–D)** show PG cells, **(E–H)** show FB cells and **(I–L)** show SG cells. Note the cycling of nuclear PER with peaks at AZT 1 (early scotophase). Abolition of daily cycling of PER after exposure to chronic LL *in vivo* **(M–X)**. Tissue samples were dissected at 6 h intervals in a 24 h period at 1, 7 13, and 19 h. **(M–P)** show PG cells, **(Q–T)** show FB cells and **(U–X)** show SG cells. Arrows in **(A,E,I,M,Q,U)** show fluorescent nuclei. Scale bars = 10 μm

Three cell types, PG, SG, and FB cells were examined for PER cycling using immunohistochemistry on day 12 after a blood meal of LD animals. All three cell types exhibited a clear daily rhythm in both abundance and cellular location of PER fluorescence (Figures 1A–D for PG cells; E–H for FB cells, and I–L for SG cells). Nuclear PER fluorescence in PG cells was intense during early scotophase at 1 h after lights-off (AZT 1) and declined in middle scotophase at 7 h after lights-off (AZT 7), indicating depletion of the fluorescent material during the scotophase (Table 1). This decrease in intensity from AZT 1 to AZT 7 was highly statistically significant (*P* < 0.01). Fluorescence intensity continued to decline during the photophase showing that PER in PG nuclei undergoes cycling during the course of a day with a peak in early scotophase. The same pattern of changes during the course of a day in nuclear fluorescence intensity was also observed in FB (Table 2; *P* < 0.01 when AZT1 was compared with AZT 7) and SG cells (Table 3; *P* < 0.05 when AZT1 was compared with AZT7). Therefore, all three cell types exhibit daily cycling of nuclear PER with peaks in early scotophase, that is synchronous in all cell types. Nuclear PER fluorescence peaked in all three cell types around the time of dusk.

**Table 1:** **PG cells (mean relative PER fluorescence ± SEM every 6 h throughout a day)**.

**PG CELLS IN 12D:12L**		
**AZT**	**Nucleus**	**Cytoplasm**
1	3508 ± 205	2263 ± 255
7	2531 ± 140	2953 ± 207
13	2621 ± 167	1914 ± 177
19	2198 ± 212	1946 ± 152
**PG CELLS IN LL**		
**Hours in a 24 h cycle**	**Nucleus**	**Cytoplasm**
1	1832 ± 151	1866 ± 175
7	1825 ± 118	1854 ± 92
13	1811 ± 190	1798 ± 120
19	1819 ± 96	1877 ± 111

N = 8 animals.

**Table 2:** **FB cells (mean relative PER fluorescence ± SEM every 6 h throughout a day)**.

**FB CELLS IN 12D:12L**		
**AZT**	**Nucleus**	**Cytoplasm**
1	2960 ± 42	2889 ± 160
7	2197 ± 112	1729 ± 98
13	2613 ± 218	2320 ± 112
19	2736 ± 220	2238 ± 130
**FB CELLS IN LL**		
**Hours in a 24 h cycle**	**Nucleus**	**Cytoplasm**
1	2307 ± 198	1500 ± 101
7	2176 ± 103	1526 ± 122
13	2238 ± 162	1490 ± 115
17	2336 ± 164	1503 ± 141

N = 8 animals.

**Table 3:** **SG cells (mean relative PER fluorescence ± SEM every 6 h throughout a day)**.

**SG CELLS IN 12D:12L**		
**AZT**	**Nucleus**	**Cytoplasm**
1	2708 ± 169	2137 ± 137
7	1301 ± 128	1099 ± 83
13	1927 ± 136	1524 ± 116
19	2373 ± 144	1567 ± 189
**SG CELLS IN LL**		
**Hours in a 24 h cycle**	**Nucleus**	**Cytoplasm**
1	1918 ± 127	1629 ± 86
7	1944 ± 188	1719 ± 93
13	1990 ± 184	1612 ± 101
19	1873 ± 125	1697 ± 94

N = 8 animals.

Cytoplasmic PER fluorescence also showed daily cycling in all three cell types, but the phase of the daily rhythm differed between cell types. In the cytoplasm of PGs (Table 1), PER fluorescence intensity also cycled. It increased to a peak at AZT 7, which was significantly higher than the immediately preceding point AZT 1 and the following point AZT 19 (*P* < 0.01 for both comparisons). These analyses reveal that when cytoplasmic PER increased to a peak, nuclear PER declined to a trough and when nuclear PER increased to a peak, cytoplasmic PER decreased to a trough. All this suggests daily cycling of PER between the two cellular compartments in PG cells. Cytoplasmic PER fluorescence also cycled in FB cells (Table 2) and SG cells (Table 3) with troughs at AZT 7, which were highly significant when compared to the preceding point AZT 1 (*P* < 0.01 for both cell types) and marginally significant when compared to the following point AZT 19 (*P* = 0.05 for either cell type). Therefore, cytoplasmic PER exhibited a daily rhythm in fluorescence intensity in FB and SG cells that was in synchrony with their rhythms in nuclear PER fluorescence intensity. Thus, regulation of movement of PER in FB and SG cells seem to differ from that in PG cells (see Discussion).

### Depletion of PER and abolition of its cycling in chronic LL *in vivo*

**Figure 2:**
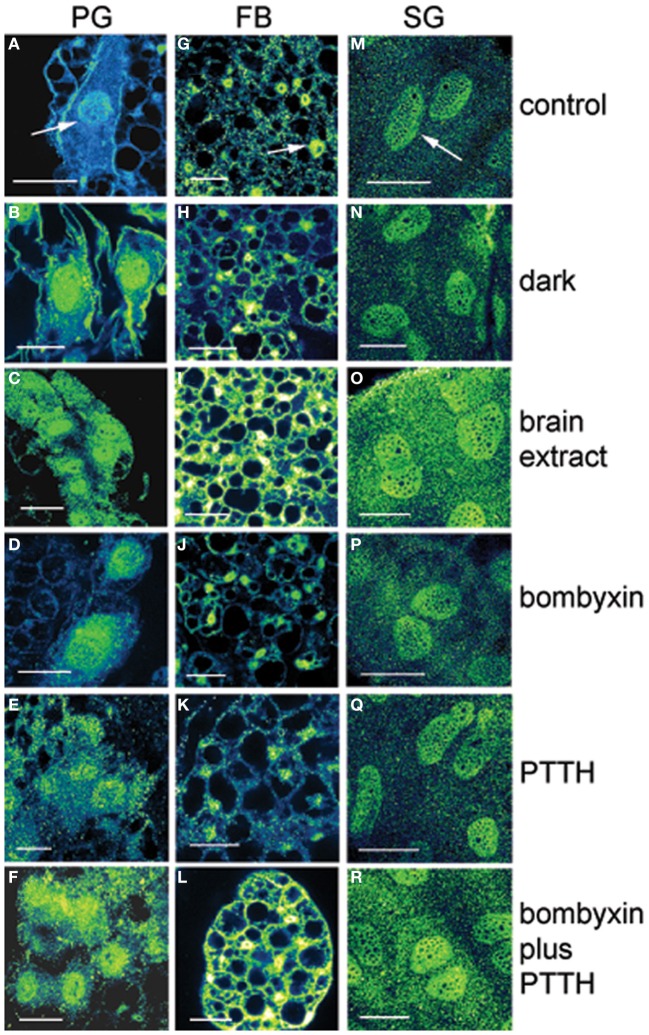
**Effects of various treatments *in vitro* on PER immunofluorescence in PG cells (A–F), FB cells (G–L), and SG cells (M–R) from arrhythmic LL animals at day 12 after feeding.** Fluorescence in shown as green/yellow. Arrows in **(A,G,M)** show nuclei. Controls were incubated in light. In PG cells note the substantial increase in nuclear immunofluorescence intensity following all treatments compared to controls. Note that transfer to dark and treatments with bombyxin or PTTH had no effect in FB and SG cells. Note that treatment with brain extract, which contains both ILP and PTTH, and combination treatment with both bombyxin (an ILP) and PTTH increased substantially both nuclear and cytoplasmic fluorescence intensity in all three cell types when compared with other treatments. Scale bars = 10 μm.

The experimental treatments described in the following sections involving transfer to darkness and the effects of neuropeptides all employ animals that had been maintained for 3 or more weeks in LL (see Methods). Therefore, it was first necessary to document the behavior of PER in the cells of these LL animals. Chronic exposure to light depleted PER by approximately 30–50% (depending on the cell type) throughout a day and abolished the daily peak of PER fluorescence in both nuclei and cytoplasm of all three cell types (Figures 1M–P for PGs; Q–T for FB; U–X for SGs). However, LL treatment did not completely eliminate PER from any cell type (see Discussion). Kruskal-Wallace analysis of the LL data (Table 1 for PGs; Table 2 for FB; Table 3 for SGs) revealed no significant variation in fluorescence intensities over the course of a day in either the nucleus or the cytoplasm in any of the three cell types. Therefore, chronic exposure to LL abolished PER rhythmicity in all three tissues. Controls for the experiments below consisted of incubation of the above cell types in light for 4 h *in vitro* (Figures 2A for PGs, G, for fat body, M, for SGs).

### PER induction in PG cells *in vitro*

#### Transfer to darkness

It is known that steroid synthesis by the PG cells becomes arrhythmic in prolonged LL and that rhythmicity is re-initiated within 4 h of transfer to darkness *in vitro* (Vafopoulou and Steel, 1998). However, it has not been shown that this effect is mediated by the action of the light cue on the clock in the PG cells. Here, we found the level of PER fluorescence in PGs transferred from LL to darkness increased rapidly in both cytoplasm and nucleus (*P* < 0.01 for both compartments) (Figure 2B) to levels comparable to the daily peak values of entrained animals (Table 4). Since the levels of PER in LL are low (Figure 2A; Table 1), we infer that transfer to darkness re-initiates production of PER and its rapid entry into to the nucleus.

**Table 4:** **Mean relative PER fluorescence ± SEM in PG, FB and SG cells following various *in vitro* treatments**.

**Treatment**	**Nucleus**	**Cytoplasm**
**PG CELLS**		
Control	1424 ± 48	1283 ± 79
Darkness	3247 ± 197	2146 ± 144
Brain extract	3012 + 168	2388 ± 158
Bombyxin	2718 ± 109	2234 ± 68
PTTH	2265 ± 79	2481 ± 105
Bombyxin plus PTTH	3494 ± 58	2866 ± 134
**FB CELLS**		
Control	2238 ± 264	1442 ± 149
Darkness	2150 ± 223	1529 ± 124
Brain extract	3084 ± 187	1952 ± 100
Bombyxin	2453 ± 272	1626 ± 134
PTTH	2469 ± 283	1481 ± 264
Bombyxin plus PTTH	3192 ± 94	1854 ± 59
**SG CELLS**		
Control	1806 ± 78	1557 ± 60
Darkness	1780 ± 130	1535 ± 79
Brain extract	2185 ± 54	1934 ± 83
Bombyxin	1893 ± 53	1465 ± 60
PTTH	1883 ± 153	1558 ± 90
Bombyxin plus PTTH	2378 ± 73	1905 ± 55

N = 8 animals.

#### Action of neuropeptides

All four neuropeptide treatments (brain extract, bombyxin, PTTH, and bombyxin plus PTTH) induced significant increases in the level of both nuclear and cytoplasmic PER fluorescence (Figures 2C–F; Table 4) (*P* < 0.01 for both nucleus and cytoplasm when compared to control LL nucleus and cytoplasm). This increase was about 2–2.5-fold dependent on the treatment. That both bombyxin and PTTH independently induced PER indicates that the signaling pathways of these two neuropeptides are involved in PER expression (see Discussion). It appears that darkness was marginally more effective than either neuropeptide (*P* = 0.05 when nuclear and cytoplasmic PER fluorescence intensities after transfer to dark were compared to the corresponding nuclear and cytoplasmic fluorescence intensities after treatment with these neuropeptides). Importantly, exposure of PG cells to both neuropeptides simultaneously (Figure 2F, Table 4), or to brain extract (Figure 2C; Table 4), induced an increase in PER fluorescence to a significantly higher level in both nuclear and cytoplasmic fluorescence intensities when these values were compared to the corresponding values of intensities in nucleus and cytoplasm of PG cells treated by either neuropeptide alone (*P* < 0.01 for all comparisons) and even exceeded the level induced by transfer to darkness (*P* < 0.05 for both comparisons). The finding that nuclear and cytoplasmic levels of PER fluorescence both increase very rapidly following all of these treatments implies swift translocation to the nucleus of newly induced PER.

### PER induction in FB and SG cells *in vitro*

#### Transfer to darkness

Neither FB nor SG cells responded to transfer to darkness with any change in the level of PER fluorescence in either nuclei or cytoplasm (Figures 2H,N respectively; Table 4) (*P* > 0.2 in all comparisons). Thus, PER levels in both cell types are insensitive to lights-off.

#### Action of neuropeptides

Treatment with bombyxin or PTTH had no effect on the level of either nuclear or cytoplasmic PER in either FB (Figures 2J,K respectively) or SG (Figures 2P,Q respectively) cells (*P* > 0.2 for all comparisons) (Table 4). However, simultaneous treatment with both bombyxin and PTTH induced significant increases in PER fluorescence in both nuclei (*P* < 0.01 for either cell type) and cytoplasm (*P* < 0.05 for either cell type) when values were compared to corresponding LL values for either cell type (Figure 2L for FB cells and Figure 2R for SG cells) Brain extract was also as effective as this combination of neuropeptides. These findings indicate that simultaneous activation of both the insulin and PTTH signaling pathways is required to cause upregulation of PER expression in FB and SG cells (see Discussion).

### PER and PDF induction in brain clock cells

**Figure 3:**
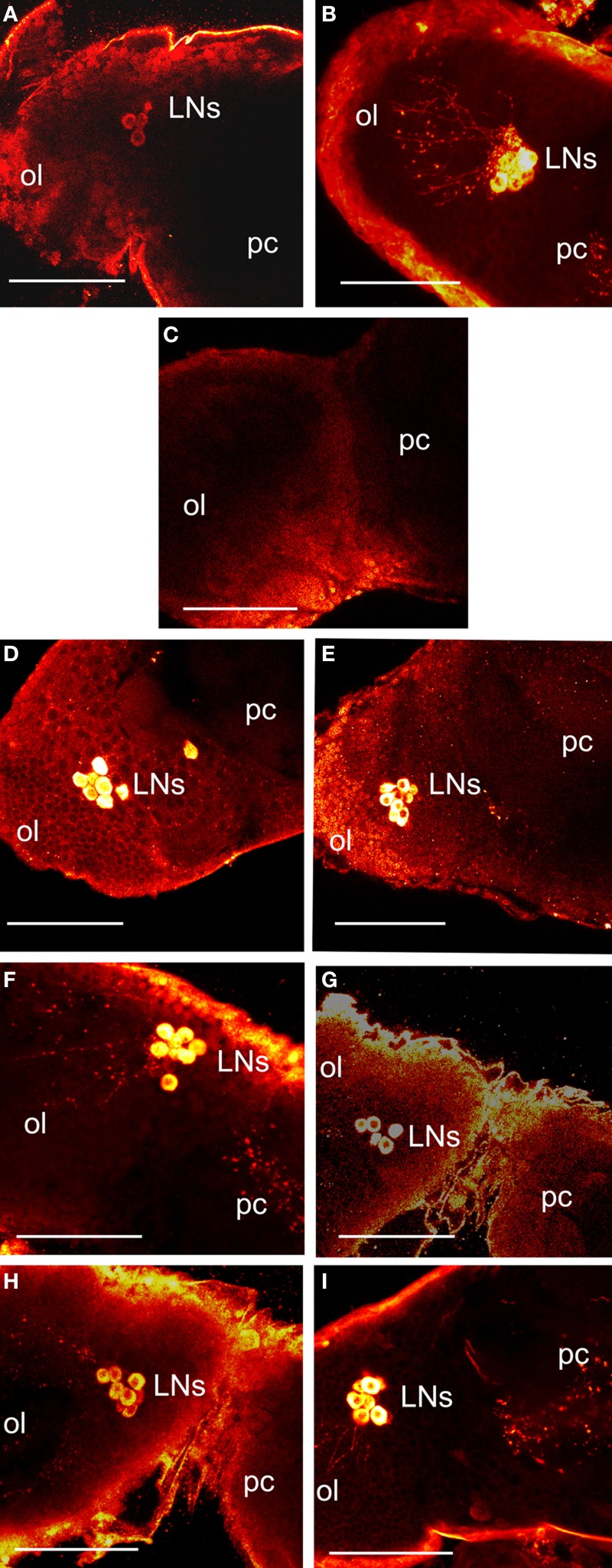
**Images of control and treated brains 12 days after a blood meal.** All images are a dorsal view of the right brain hemisphere. **(A–C)** show *in vivo* brains. Control scotophase brains from entrained animals in 12L:12D stained with anti-PER **(A)** or anti-PDF **(B)**. Fluorescence is shown as yellow/white. Images show the lateral clock neurons (LNs) at the junction of protocerebrum (pc) and optic lobe (ol) and their axonal projections. **(C)** a brain from animals transferred to LL for 3 weeks and stained with anti-PDF; note absence of PDF fluorescence in LNs. LL brains similarly stained with anti-PER also showed no staining in the LNs (not shown). **(D–I)** show LL brains incubated *in vitro.* **(D–E)** show brains stained with anti-PER. **(D)** LL brain transferred to darkness. **(E)** brain challenged with bombyxin. Note induction of PER (compare with **C**). **(F–I)** show brains stained with anti-PDF. **(F)** brain transferred to darkness. **(G)** brain challenged with bombyxin. **(H)** brain challenged with anti-PTTH. **(I)** brain challenged with bombyxin plus PTTH. Scale bars = 10 μm.

Vafopoulou et al. (2010) showed that PER fluorescence in *Rhodnius* brain clock cells showed circadian cycling in 12L:12D; the main group of clock cells (lateral clock neurons; LNs) were also filled with PDF, which enabled tracing of their axons. Figure 3A shows the location of LNs stained with anti-PER (Figure 3A) and anti-PDF (Figure 3B) on the border of the optic lobe and protocerebrum of a control scotophase animal entrained in 12L:12D at day 12 after a blood meal. We have shown previously (and discussed in depth) that PER fluorescence in LNs is exclusively cytoplasmic (Vafopoulou et al., 2010; Vafopoulou and Steel, 2012a,b). Here, we first examined the effect of transfer of 12 such animals to LL for 3 weeks on levels of PER and PDF in LNs *in vivo*. All brains were completely devoid of PER fluorescence and 10 of the 12 were also devoid of PDF fluorescence. Two of the 12 brains showed trace levels of PDF in their somata and none in their axons. Figure 3C shows an LL brain stained with anti-PDF in which PDF fluorescence in LNs is reduced to background levels. PER fluorescence in LNs is similarly reduced to background levels (not shown). Thus, chronic LL completely abolishes PER in brain clock cells and PDF is abolished or drastically reduced.

Brains were excised from these LL animals, incubated *in vitro* and challenged with transfer to darkness or with neuropeptides.

#### Transfer to darkness

A group of 15 LL brains was transferred to darkness and PER fluorescence was examined after 4 h incubation *in vitro.* In all 15 brains, PER fluorescence re-appeared in the somata of LNs (Figure 3D). In addition, PDF fluorescence was also restored and had migrated down the axons in all brains (Figure 3F). Therefore, transfer to darkness induced both PER and PDF fluorescence within 4 h.

#### Action of neuropeptides

Groups of 15 LL brains were incubated *in vitro* with either bombyxin or PTTH or both neuropeptides together and then stained for PER (Figure 3E) or PDF (Figures 3G–I). All neuropeptides induced strong PER fluorescence in LNs (Figure 3E shows incubation with bombyxin; images from incubations with PTTH or bombyxin plus PTTH are not shown). All neuropeptides also induced strong PDF fluorescence (Figure 3G, incubation with bombyxin; Figure 3H, incubation with PTTH; Figure 3I, incubation with bombyxin plus PTTH). The pattern and intensity of induced fluorescence were closely similar to that normally seen *in vivo* in scotophase brains from animals entrained in 12L:12D, and similar to that induced by transfer to darkness (Table 5). Of particular interest is the finding that incubation with both bombyxin and PTTH together induced significantly greater fluorescence in LNs than did incubation with either neuropeptide alone (*P* < 0.01 for both comparisons). Therefore, both signaling pathways are involved in the expression of both PER and PDF (see Discussion).

**Table 5:** **Mean relative PER fluorescence ± SEM in LNs following various *in vitro* treatments**.

**Treatment**	**Cytoplasm**
**LNs**	
Control	646 ± 12
Darkness	1802 ± 51
Brain extract	1933 ± 28
Bombyxin	1907 ± 38
PTTH	1690 ± 59
Bombyxin plus PTTH	2011 ± 44

N = 15 animals.

## Discussion

### Induction of PER by neurohormones and photic cues in various cell types

The present paper demonstrates that the neurohormones bombyxin (an ILP), and PTTH play an important role in the regulation of the clock protein PER in various tissue types of *Rhodnius*. Insulin and members of the family of insulin/insulin-like growth factors target a wide variety of tissues and exhibit a plethora of effects such as mediation of nutrition on cell growth, development, longevity, senescence and metabolic homeostasis of insects (reviews by Wu and Brown, 2006; Hunt et al., 2007; Nässel and Winther, 2010; Shingleton, 2010; Teleman, 2010, and articles in this Research Topic). On the other hand, the only documented target of PTTH is the PGs and knowledge of its signaling pathway derives exclusively from studies of its ecdysteroidogenic action on PGs. These two seemingly unrelated signal transduction systems are both involved in the regulation of PER. Moreover, this regulation appears to be achieved by convergence of the signaling pathways for these hormones as discussed below. The details of this convergence vary with the cell type under study. Consequently, we introduce the information of PER regulation first in the context of the differing characteristics of PER expression in the different cell types, before presenting a more generalized interpretation.

All tissues studied here (brain, PG, FB, SG) are known to play roles in circadian timekeeping and to express PER cyclically. But these roles differ greatly between tissues, and thus so does the significance of the action of neurohormones on PER expression in them.

The molecular mechanism of circadian timekeeping was first elucidated in specific neurons of the brain of *Drosophila*. Briefly, “clock genes” (such as *per*) are cyclically transcribed as a result of indirect stimulation and inhibition by feedback from their own protein products (e.g., PER) within a complex molecular oscillator (MO) (review by Hardin, 2011). PER and *per* play critical, central roles and are regarded as canonical components of the MO. Thus, circadian cycling of PER is viewed as a necessary feature of the MO. All four tissue types examined here exhibited robust daily cycling of PER in both abundance and cellular localization, with peak nuclear localization in the scotophase of all types.

The brain clock system of *Rhodnius* (Vafopoulou et al., 2010; Vafopoulou and Steel, 2012a) is very similar in organization to that of *Drosophila* (reviews by Helfrich-Förster, 2003, 2005; Nitabach and Taghert, 2008). One group of clock cells in *Drosophila* (LNs) is photosensitive (Klarsfeld et al., 2011) and is traditionally regarded as the pacemaker (Vossall and Young, 1995). *Rhodnius* LNs exhibit circadian cycling of PER (Vafopoulou et al., 2010) and also express the neuropeptide PDF, which fills their axonal projections (Vafopoulou et al., 2007, 2010; Vafopoulou and Steel, 2012a). PDF has been considered an output protein of the brain clock (review by Taghert and Shafer, 2006; Helfrich-Förster, 2009). PDF-filled axons project to and make intimate associations with axonal projections from both the ILP (Vafopoulou and Steel, 2012b) and PTTH (Vafopoulou et al., 2007) neurons, providing a neural pathway for driving the known rhythmic release of these neuropeptides into the circulation (Vafopoulou and Steel, 1996a, 2002). Here, we found that chronic LL completely abolished fluorescence due to both PER and PDF in the LNs. Transfer of brains to darkness *in vitro* restored both PER and PDF within 4 h. We infer that *Rhodnius* LNs are photosensitive and that a light cue induces both PER and PDF. This conclusion implies that *pdf* transcription resumes when the MO is restarted by a cue from either light or a neurohormone, i.e., *pdf* is a clock-controlled gene. The effects of the neurohormones bombyxin and PTTH on the brain clock *in vitro* were particularly remarkable. Both hormones promptly induced both PER and PDF fluorescence to levels typical of normal scotophase animals *in vivo*, i.e., both hormones acted on the brain similarly to darkness. Since the brain clock itself controls the rhythmic release of both these hormones, these findings indicate a “temporal feedback” whereby the released hormones act back on the clock in the LNs to affect PER expression. We are unaware of any prior evidence that brain neurohormones act back on the brain clock in insects. Clearly, the brain clock is responsive not only to external light signals but also to internal hormonal signals as well (see Discussion below).

The PG cells of *Rhodnius* are well documented peripheral clocks (Vafopoulou and Steel, 1992, 1998) that rhythmically synthesize ecdysteroids. Rhythmic synthesis persists *in vitro* (Vafopoulou and Steel, 1992) and can be reinitiated by transfer of arrhythmic LL PGs *in vitro* to darkness (Vafopoulou and Steel, 1998). PG cells were severely depleted of PER and its daily rhythmicity was abolished by chronic exposure to LL. Transfer of these PGs to darkness *in vitro* resulted in prompt and massive increases in both nuclear and cytoplasmic PER in PG cells. Continued examination of these tissues for several days *in vitro* showed that induced PER exhibited a free-running rhythm in abundance and nuclear location (unpublished). Thus, the present data show that re-initiation of the ecdysteroid synthesis rhythm is mediated by the action of the light cue on a PER-based MO in the PG cells. Bombyxin and PTTH are known to act as stimulators of ecdysteroid synthesis in PGs of *Rhodnius*, with bombyxin being less effective than PTTH (Vafopoulou and Steel, 1997). The action of PTTH is believed to be control of the phase of the PG clock (Pelc and Steel, 1997). Here, we found that both neurohormones (administered separately *in vitro*) induced substantial increases in both nuclear and cytoplasmic PER fluorescence in PG cells, but not to the levels induced by darkness or to those seen *in vivo*. However, when both peptides were administered together, the resulting levels of PER fluorescence exceeded both those induced by darkness and those seen *in vivo* (see Discussion below).

The status of SG and FB cells as circadian clocks is much less clear than the above. Here, we found that both cell types exhibit daily cycling of PER *in vivo*, but cycling does not persist *in vitro* (unpublished), implying that these cell types are oscillators that are driven by cues received *in vivo* and are not self-sustained clocks. This view is supported by the finding that cytoplasmic and nuclear PER fluorescence in FB and SG cells cycle in synchrony with each other, in contrast to the asynchronous behavior seen in brain LN and PG cells. Such synchrony is not consistent with daily migration of PER between cytoplasm and nucleus. Nevertheless, PER levels in these cell types were drastically depleted in chronic LL, as in the other tissue types. But, unlike the other tissue types, transfer to darkness *in vitro* did not restore PER fluorescence in either cytoplasm or nucleus. We conclude that light cues have no effect on PER in these tissues. Further, neither treatment with bombyxin nor PTTH *in vitro* induced PER fluorescence. However, treatment with both peptides simultaneously did increase PER fluorescence in both nuclei and cytoplasm in both SG and FB cells. Thus, induction of PER expression in these cells requires simultaneous exposure to both neuropeptides to induce PER expression (see Discussion below).

### Potential mechanism of induction of PER by neurohormones

Both bombyxin and PTTH rapidly induce PER when applied *in vitro* to either brains or PGs. A direct effect of bombyxin and PTTH on the clock cells is probable, most obviously in the case of their action on PGs, in which all cells are clock cells (Vafopoulou and Steel, 1998). In the brain, the possibility of hormone action on interneurons cannot be excluded. Nevertheless, in both tissues the actions must be mediated via cellular signaling pathways, whether within the clock cells themselves or within neurons that control them.

Signal transduction of insulin is activated by binding of insulin to its cognate receptor which leads to a cascade of phosphorylations of a series of intracellular signal transducer enzymes such as the phosphoionositide kinase 3 (PI3 K), which in turn activates a cytosolic serine-threonine kinase (Akt), also called protein kinase B (PKB). This pathway interacts with the TOR pathway (target of rapamycin) through activation by phosphorylation of TOR which regulates functions such as cell growth, differentiation and survival (e.g., Nojima et al., 2003; Tokunaga et al., 2004). Subsequently, Akt inhibits by phosphorylation the nuclear translocation of the transcription factor Forkhead fox O (FOXO); FOXO regulates metabolism, growth, energy homeostasis, stress responses, life span and senescence (for references see Puig and Mattila, 2011). It is reasonable to assume that the action of bombyxin on *Rhodnius* PGs is mediated by a typical insulin signaling pathway as has been shown in other insect systems (e.g., Nijhout et al., 2007; Nagata et al., 2008).

There is currently no evidence that the signaling pathways for insulin or PTTH influence PER expression. However, there is evidence that the insulin signaling pathway affects another canonical clock protein, TIMELESS (TIM) (Zheng and Sehgal, 2010). In the *Drosophila* brain, increased TOR signaling, a pathway that interacts with the insulin pathway, resulted in TIM accumulation in the nuclei of small lateral ventral clock neurons (LN_v_s), a subgroup of LNs (Zheng and Sehgal, 2010). In addition, genetic manipulation of the expression of Akt and TOR in the LN_v_s, which control the activity rhythm of *Drosophila,* revealed that both enzymes are involved in the regulation of period length of the activity rhythm (Zheng and Sehgal, 2010). This relationship could be reciprocal resulting in a functional cross-talk between components of the clock and insulin signaling as found in a human cell line by Zhang et al. (2009). In this study, use of a large scale screening of individual small interfering RNAs, a large number of knockdown genes and protein interaction network analysis identified a strong relationship between the insulin signaling pathway and the circadian clock. In this system, downregulation of multiple components of the insulin signaling pathway changed the period length of the clock. Conversely, transcription of multiple components of the insulin signaling pathway was regulated by the clock. Therefore, the insulin transduction system and the molecular clock interact in a variety of animal systems.

With respect to PTTH, its only documented action is on PGs. The classical PTTH signaling pathway is unrelated to the insulin signaling pathway. It involves the binding of PTTH to its cognate receptor, Torso, a receptor tyrosine kinase (Rewitz et al., 2009b) which initiates an extensive signaling transduction network (Rewitz et al., 2009b; Marchal et al., 2010) that is mediated by an increase in intracellular in Ca^++^ and an increase Ca^++^/calmodulin-dependent cAMP (Smith et al., 1984, 1985; Gu et al., 1998, 2010), which results in a cascade of protein phosphorylations and leads to translation of several enzymes involved in steroidogenesis (review by Marchal et al., 2010). In addition, a recent quantitative phosphoproteomics approach in *Manduca sexta* revealed involvement of PTTH in the phosphoryation of many proteins unrelated to those involved in the classical PTTH signaling pathway (Rewitz et al., 2009a). These proteins regulate several different signaling pathways and include, among others, rate limiting enzymes involved in transcription and translation. These effects are seen not only in PGs but in a variety of other tissues suggesting that PTTH participates in several signaling pathways and in multiple cell types (Rybczynski et al., 2009). The present finding that PTTH induces PER is the first evidence of involvement of PTTH in regulation of a specific cellular activity that does not occur solely in PGs and is not directly related to ecdysteroidogenesis.

### Synergism between ILP and PTTH in induction of PER

The present findings broadly illustrate that bombyxin and PTTH are both able to induce PER in a variety of different cell types. But in some cell types they act independently (brain, PGs), in some they act additively (PGs) and in others are both required simultaneously (SGs and FB). It is therefore possible that the signaling pathways for these hormones may possess varying degrees of convergence in different cell types. There is strong evidence of convergence of the two pathways in PGs, which could explain the synergistic action of the two hormones on PER. Recent studies on PTTH-mediated ecdysteroidogenesis in *Bombyx* PGs revealed the existence of a second PTTH signaling pathway that involves the PI3K/Akt/TOR sequence (Gu et al., 2010, 2011, 2012). Bovine insulin also stimulated PGs by the PI3K/Akt/TOR signaling sequence (Gu et al., 2009, 2012). Therefore, both insulin and PTTH signaling pathways converge at the level of PI3K/Akt/TOR sequence. In addition, the ribosomal S6 kinase (S6K), a downstream component of the TOR pathway, regulates glycogen synthase kinase 3β (GSK3β) along with Akt. GSK3β is a key enzyme in the regulation of clock proteins in both mammals (e.g., Iitaka et al., 2005; Yin et al., 2006; Sahar et al., 2009; Spengler et al., 2009) and *Drosophila* (Martinek et al., 2001). Interestingly, S6K and its ribosomal protein substrate S6 are both downstream targets of the classical PTTH signaling pathway that controls ecdysteroidogenesis in *Manduca* (Song and Gilbert, 1997). Rapamycin, a specific inhibitor of TOR, also inhibited the PTTH-mediated phosphorylation of S6K and ecdysteroidogenesis (Song and Gilbert, 1994, 1995; Gu et al., 2012). Therefore, the insulin and PTTH signaling pathways could also converge at the level of S6K. Therefore, both pathways converge at multiple points. We infer that there is extensive cross talk between insulin and PTTH signaling involved in the regulation of PER in PGs.

By contrast, when SG or FB cells were exposed to both bombyxin and PTTH there was a significant increase in PER in both nuclei and cytoplasm, even though these cells did not respond at all to treatments with either peptide alone. This finding shows that PER expression in cells that are peripheral oscillators is only elicited by simultaneous stimulation by ILP and PTTH and suggests that both signaling pathways, when activated, can act cooperatively to stimulate PER production in peripheral oscillators. This response would enable these peripheral oscillators to orchestrate their internal activities with respect to circadian time and also to coordinate these activities with other cells that are also responsive to both hormones.

### Implications for temporal order

The present findings show that rhythmicity in brain neurohormones can no longer be explained solely in terms of their release being driven by a brain clock system that is entrained by light cues from the external world. We have shown that the release of neurohormones acts back on the brain to influence the behavior of PER in the MO of the clock cells that drive these rhythms. The precise significance of this “temporal feedback” remains unknown, but could serve as a mechanism that helps to stabilize the daily timing of release of various rhythmic neurohormones, which would ensure synchrony in the rhythmic responses of target tissues. Even the further downstream hormone rhythms such as the ecdysteroid circadian rhythm have been found to participate in this temporal feedback, since daily shuttling of the EcR was previously reported (Vafopoulou and Steel, 2006) in both the brain neuroendocrine cells and in the clock cells themselves in the brain. Consequently, the brain clock cells are receiving rhythmic signals from at least three circulating hormones (ILP, PTTH, and ecdysteroids) and therefore appear to be integrating external light cycle information with internal information from hormone rhythms. The complexity and intricacy of these interactions between hormone rhythms and the clock system attests to the importance of precisely regulated endocrine rhythms in animal physiology. We have discussed previously (Steel and Vafopoulou, 2006) that tightly organized rhythmicity in the endocrine system is essential to the appropriate orchestration of daily events within cells and also to the synchrony of the various tissues and organs within the animal both with each other and with the external world.

### Conflict of interest statement

The authors declare that the research was conducted in the absence of any commercial or financial relationships that could be construed as a potential conflict of interest.
